# QuickStats

**Published:** 2013-04-05

**Authors:** Jiaquan Xu

**Figure:**
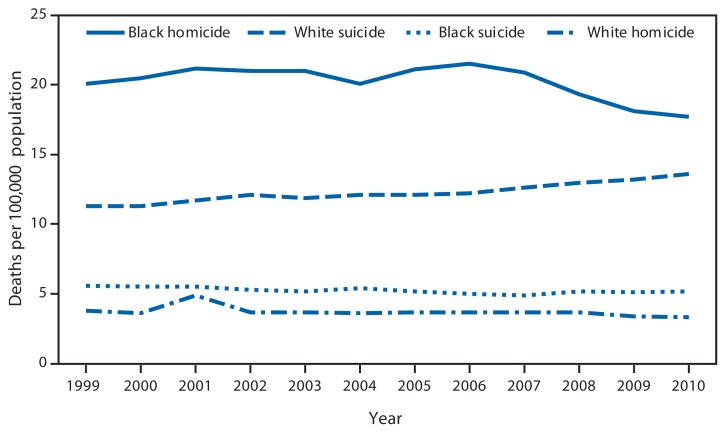
Annual Age-Adjusted Death Rates*^†^ for Suicide and Homicide, by Black or White Race — United States,^§^ 1999–2010 * Deaths are coded as *U03, X60–X84, and Y87.0 for suicide, and *U01–*U02, X85–Y09, and Y87.1 for homicide, as underlying causes of death, according to the *International Classification of Diseases 10th Revision*. Rates include deaths related to the events of September 11, 2001. ^†^ Rates have been revised by using populations enumerated as of April 1, for 2000 and 2010, and intercensal estimates as of July 1 for all other years. Therefore, the rates might differ from those published previously. ^§^ U.S. residents only.

From 1999 to 2010, annual age-adjusted homicide death rates for blacks were at least four times the rates for whites. In contrast, suicide rates for whites were twice as high as the rates for blacks. From 1999 to 2010, homicide death rates decreased 13.2% among whites, from 3.8 deaths per 100,000 population to 3.3, and suicide rates increased 20.4%, from 11.3 deaths per 100,000 population to 13.6. Among blacks, homicide death rates increased 7.0%, from 20.1 deaths per 100,000 population in 1999 to 21.5 in 2006, then decreased 17.7%, from 21.5 deaths per 100,000 population in 2006 to 17.7 in 2010. Suicide rates decreased 7.1% among blacks, from 5.6 deaths per 100,000 population in 1999 to 5.2 in 2010.

**Source:** National Vital Statistics System. Mortality public use data files, 1999–2010. Available at http://www.cdc.gov/nchs/data_access/vitalstatsonline.htm.

